# Prevalence of and risk factors for anxiety and depression in Chinese patients with unruptured intracranial aneurysms treated by endovascular intervention

**DOI:** 10.1186/s12888-020-02834-3

**Published:** 2020-09-03

**Authors:** Xiao-Dong Zhai, Jia-Xing Yu, Yong-Jie Ma, Si-Shi Xiang, Gui-Lin Li, Chuan He, Peng Hu, Hong-Qi Zhang

**Affiliations:** 1 Department of Neurosurgery, Xuanwu Hospital, No. 45 Changchun Street, Xicheng District, Beijing, China; 2 China International Neuroscience Institute (China-INI), Beijing, China

**Keywords:** Unruptured intracranial aneurysms, Endovascular intervention, Anxiety, Depression, Prevalence, Risk factors

## Abstract

**Background:**

Studies on anxiety and depression in unruptured intracranial aneurysm (UIA) patients after treatment via endovascular intervention are rare and controversial. We aimed to explore the prevalence of anxiety and depression among Chinese patients with UIAs treated by endovascular intervention and to identify which factors contribute to the development of these symptoms.

**Methods:**

We performed a cross-sectional study on anxiety and depression in patients who underwent endovascular treatment for UIAs using the Hospital Anxiety and Depression Scale (HADS). The demographic, clinical and radiological data for all patients were retrospectively collected from the aneurysm database and medical records. Moreover, we utilized data from a large sample of 200 UIA patients and multivariate logistic regression analysis to investigate the risk factors for anxiety and depression in these patients. Candidate variables with *P* values less than 0.20 in univariate analysis were included in the multivariate logistic regression analysis.

**Results:**

Two hundred patients returned completed questionnaires in this study. Of these 200 patients, 34 (17.0%) suffered from anxiety and 31 (15.5%) suffered from depression 30.67 ± 8.6 months after being discharged. The multivariate analysis results indicated that shorter sleep times were statistically significantly associated with depression (OR = 1.62, 95% CI: 1.14 ~ 2.29, *P* = 0.007, Adjusted *P* = 0.02). .

**Conclusion:**

The prevalences of anxiety and depression in UIA patients treated by endovascular intervention were 17.0 and 15.5%, respectively. Shorter sleep times were significantly associated with depression. Our findings provide evidence for the clinical and psychological management of these patients.

## Background

Unruptured intracranial aneurysm (UIA) is a common disease with a prevalence of approximately 7% in Chinese adults aged 35 to 75 years [1]. In addition, advances in high-resolution imaging technologies and the increasing availability of these methods have led to a higher number of incidentally detected UIAs [1, 2]. Once an aneurysm ruptures, it typically causes subarachnoid hemorrhage (SAH) and sequelae, resulting in significant morbidity and mortality [3, 4]. Therefore, identifying and treating UIAs with a high propensity for rupture is critical for both neurosurgeons and neuroradiologists.

Evidence suggests that anxiety and depression symptoms are commonly associated with aneurysm disease, and both are important determinants of health-related quality of life in such patients [5, 6]. The threat of rupture becomes the most obvious cause of preoperative anxiety and depression in patients with UIA [5, 7, 8]. In recent years, endovascular treatment has become a main strategy for treating for UIAs as a result of its superiority over microsurgery in both morbidity and mortality [9]. However, studies on anxiety and depression in UIA patients after treatment via endovascular intervention are rare and controversial [5, 10, 11].

We aimed to explore the prevalence of anxiety and depression among Chinese UIA patients treated by endovascular intervention and to identify which factors contribute to the development of these symptoms.

## Methods

### Study design and participants

A cross-sectional study was performed. We retrospectively reviewed the hospital database for all consecutive UIA patients who underwent endovascular treatment in the Department of Neurosurgery, Xuanwu Hospital of Capital Medical University, from January 1, 2015, to May 31, 2017. The inclusion criteria were as follows: (1) diagnosed by digital subtraction angiography (DSA) and treated with endovascular intervention; and (2) aged 18 to 75 years. The exclusion criteria were as follows: (1) had traumatic, mycotic, bacterial, dissecting or fusiform aneurysms; (2) had a history of SAH or cerebral hemorrhage due to aneurysms or other causes; (3) died during the follow-up period; and (4) had a history of treatment for intracranial aneurysms (including microsurgical or endovascular treatment) or who underwent retreatment due to the incomplete obliteration before the follow-up. Ultimately, a total of 300 patients were included in this study.

### Assessment instrument

The Hospital Anxiety and Depression Scale (HADS) is a promising tool for identifying and quantifying depression and anxiety symptoms in physically ill patients [12, 13]. Therefore, the HADS was used to assess anxiety and depression symptoms in UIA patients. The HADS is composed of seven items related to anxiety symptoms and seven related to depression symptoms, for 14 items in total [13, 14]. All 14 items were answered by the patients using a four-point Likert scale, with the aim to assess their subjective experience with anxiety and depression symptoms. In this study, anxiety and depression were dichotomized based on the recommended threshold (equal to or above 8 points), with a sensitivity and specificity in the range of 0.70 to 0.90 [15]. The content of the questionnaire was mainly composed of the HADS, which aimed to assesses participation in the presence of depressive and anxiety symptoms, for the previous 4 weeks. In addition, the questionnaire includes important items such as average daily sleep time, educational level, ratio of treatment costs to annual household income and physical exercise level within the last month. A combined online questionnaire was sent to the patients who were willing to respond.

### Data collection

We conducted a telephone follow-up of the 300 patients who met the inclusion and exclusion criteria; of these patients, 270 were successfully followed up, for a follow-up rate of 90.0%. We sent the combined online questionnaire to 228 patients who were willing to respond. If we received no response 7 days after sending the first online questionnaire, we sent another copy of the same online questionnaire to the patient, and the deadline for receiving the completed questionnaire was 2 weeks after the first attempt. Eventually, 200 patients returned a completed questionnaire within the set time, accounting for 66.7% of all 300 UIA patients. The demographic, clinical and radiological data for all patients were retrospectively collected from the aneurysm database and medical records. The duration since treatment was defined as the time from treatment to the follow-up. A good neurological outcome was defined as a modified Rankin Scale (mRS) score of 2 or less. In addition, mRS progression at discharge was defined as an increase in the mRS score at the time of discharge compared to the preoperative score. Due to the high representativeness, the ratio of treatment costs to annual household income is recommended as an evaluation of the treatment-related financial burden affecting symptoms of anxiety and depression. Radiological follow-up examinations included magnetic resonance angiography (MRA), computed tomography angiography (CTA) or DSA.

### Statistical methods

Categorical variables were compared between the 2 groups using the Pearson χ2 test, continuity correction, and Fisher’s exact 2-tailed test. Continuous variables were compared between groups using Student’s t-test. Interval data are reported as the mean ± standard deviation, and nominal data are expressed as absolute numbers and valid percentages. Data were tested for normality by making P-P and Q-Q plots. A multivariate logistic regression analysis was used to identify the association of the described variables with anxiety and depression in patients. Candidate variables with *P* values less than 0.20 in univariate analysis were included in the multivariate logistic regression analysis. Statistical significance was defined as *P* < 0.05. To account for the multiple testing, the *P*-values were corrected by false discovery rate (FDR). Statistical analysis was performed using SPSS Statistics version 24.0 software (IBM Corp., Armonk, New York, USA).

## Results

### Demographic, clinical and radiological characteristics

Two hundred patients returned completed questionnaires. All patient information is shown in Table 1 and Table 2. Of the 200 patients, 117 were female, and 83 were male; their mean age was 55.2 ± 9.48 years (range 23–74 years). They had a total of 245 aneurysms (161 patients had one aneurysm, 34 had two, 4 had three, and one had four), and the majority of aneurysms occurred in the anterior circulation (81.5%) (Table 2). A total of 160 (80%) patients underwent stenting or flow diversion (FD), and 40 patients (20%) underwent simple coiling. No technical failures were observed in our cohort, and 8 patients experienced postoperative ischemia-related complications. Among them, one patient with severe postoperative ischemic complications had an mRS of 5 at discharge, and the other patients exhibited good postoperative neurological outcomes (mRS = 0–2). All patients presented with good neurological outcomes (mRS = 0–2) at follow-up. Ten patients (5%) showed mRS progression at discharge compared with the preoperative mRS score. For all 200 patients, the duration since treatment ranged between 18 and 45 months, with an average of 30.67 ± 8.6 months. Radiological follow-up was available for 167 patients, with a mean follow-up period of 30.76 ± 8.43 months; 7 patients experienced incomplete obliteration and had not received further treatment at follow-up.

**Table 1:** Demographic characteristics of UIAs patients

Characteristics		Value (n, %)
Age,years,mean ± SD		55.2 ± 9.48
Gender (Female)		117 (58.5%)
Hypertension		108 (54.0%)
Diabetes		17 (8.5%)
Heart disease		25 (12.5%)
^1^ICVD history		37 (18.5)
Smoking		23 (11.5%)
Physical exercise	At least once a week	140 (70.0%)
	0 time a week	60 (30.0%)
Sleep time		6.88 ± 1.19
Education level, years	≤12	131 (65.5%)
	>12	69 (34.5%)
Anxiety		34 (17.0%)
Depression		31 (15.5%)
^2^Ratio	0–100%	133 (66.5%)
	>100%	67 (33.5%)

^1^ICVD represents “Ischemic cerebrovascular disease”, ^2^Ratio represents “Ratio of treatment cost to annual household income”

**Table 2:** Clinical and radiological characteristics of UIA patients

Characteristics		Value(n, %)
Diagnosis to treatment time	0–3 months	158 (79.0%)
	>3 months	42 (21.0%)
Intervention materials	Simple coiling	40 (20.0%)
	Stent or ^1^FD	160 (80.0%)
mRS progression at discharge	No	190 (95.0%)
	Yes	10 (5.0%)
Treatment-related complications		8 (4.0%)
Duration since treatment	≤30 months	101 (50.5%)
	>30 months	99 (49.5%)
Incomplete obliteration		7/167 (4.19%)
Multiple aneurysms		39 (19.5%)
Location	Anterior circulation	163 (81.5%)
	Posterior circulation	32 (16.0%)
	Both	5 (2.5%)

^1^FD represents “Flow diversion”

### Prevalence and risk factors of anxiety and depression

As shown in Table 2, of the 200 UIA patients, 34 (17.0%) suffered from anxiety and 31 (15.5%) suffered from depression 30.67 ± 8.6 months after being discharged. Table 3 summarizes the univariate analysis of the determinants of anxiety and depression. The independent variables with *P* values less than 0.20 in the univariate analysis were included in the multivariate logistic regression analysis. Therefore, anxiety-related variables, including education level, hypertension, ischemic cerebrovascular disease history, location of aneurysm, sleep time and ratio of treatment costs to annual household income, were included; depression-related variables, including gender, sleep time, and ratio of treatment costs to annual household income, were included. Table 4 presents the explanatory variables that were significantly associated with anxiety and depression. The multivariate analysis results indicated that shorter sleep times were statistically significantly associated with depression (OR = 1.62, 95% CI: 1.14 ~ 2.29, *P* = 0.007, Adjusted *P* = 0.02). Treatment cost exceeding the annual household income (OR = 2.42, 95% CI: 1.08 ~ 5.41, *P* = 0.03) and shorter sleep times (OR = 1.51, 95% CI: 1.06 ~ 2.14, P = 0.02) were significantly associated with anxiety, however, this association was not statistically significant after FDR correction (*P* = 0.11 and 0.14, respectively).

**Table 3:** Univariate analysis of the anxiety and depression in UIA patients

Variables	Anxiety	non-Anxiety	*P*-value	Deperssion	non-Depression	*P*-value
Age,years,mean ± SD	54.9 ± 8.88	55.25 ± 9.62	0.86	55.26 ± 9.40	55.19 ± 9.52	0.97
Gender (male)	16 (47.1%)	67 (40.4%)	0.47	9 (29.0%)	74 (43.8%)	**0.13**
Hypertension	23 (67.6%)	85 (51.2%)	**0.08**	19 (61.3%)	89 (52.7%)	0.44
Diabetes	3 (8.8%)	14 (8.4%)	0.99	3 (9.7%)	14 (8.3%)	0.99
Heart disease	4 (11.8%)	21 (12.7%)	0.99	3 (9.7%)	22 (13..0%)	0.83
^1^ICVD history	9 (26.5%)	28 (16.9%)	**0.19**	7 (22.6%)	30 (17.8%)	0.52
Smoking	5 (14.7%)	18 (10.8%)	0.73	3 (9.7%)	20 (11.8%)	0.97
Physical exercise	23 (67.6%)	117 (70.5%)	0.74	19 (61.3%)	121 (71.6%)	0.25
Sleep time	6.53 ± 1.21	6.95 ± 1.18	**0.06**	6.29 ± 1.22	6.99 ± 1.16	**0.003**
Education level, years ≤12 years	26 (76.5%)	105 (63.3%)	**0.14**	22 (71.0%)	109 (64.5%)	0.49
^2^Ratio > 100%	17 (50.0%)	50 (30.1%)	**0.03**	14 (45.2%)	53 (31.4%)	**0.14**
Diagnosis to treatment time ≤ 3 months	27 (79.4%)	131 (78.9%)	0.99	25 (80.6%)	133 (78.7%)	0.82
mRS progression at discharge	3 (8.8%)	7 (4.2%)	0.49	3 (9.7%)	7 (4.1%)	0.39
Intervention materials (Stent or ^3^FD)	29 (85.3%)	131 (78.9%)	0.40	26 (83.9%)	134 (79.3%)	0.56
Treatment-related complications	3 (8.8%)	5 (3.0%)	0.27	1 (3.2%)	7 (4.1%)	0.99
Duration since treatment	31.50 ± 7.82	30.49 ± 8.77	0.54	30.06 ± 8.06	30.78 ± 8.72	0.67
Incomplete obliteration	2 (6.7%)	5 (3.6%)	0.81	2 (7.7%)	5 (3.5%)	0.66
Multiple aneurysms	7 (20.6%)	32 (19.3%)	0.99	8 (25.8%)	31 (18.3%)	0.34
Remain untreated aneurysm	1 (2.9%)	9 (5.4%)	0.86	1 (3.2%)	9 (5.3%)	0.96
Location			**0.04**			0.32
Anterior circulation	23 (67.6%)	140 (84.3%)		24 (77.4%)	139 (82.2%)	
Posterior circulation	9 (26.5%)	23 (13.9%)		5 (16.1%)	27 (16.0%)	
Both	2 (5.9%)	3 (1.8%)		2 (6.5%)	3 (1.8%)	

^1^ICVD represents “Ischemic cerebrovascular disease”, ^2^Ratio represents “Ratio of treatment cost to annual household income” ^3^FD represents “Flow diversion”

**Table 4:** Multivariable analysis of the anxiety and depression in UIA patients

	Variables	OR (95%CI)	*P*-value	Adjusted *P*-value
Anxiety	Hypertension	1.99 (0.86 ~ 4.61)	0.11	0.19
	^1^ICVD history	2.03 (0.77 ~ 5.30)	0.15	0.21
	Education level, years≤12 years	1.34 (0.54 ~ 3.35)	0.53	0.53
	^2^Ratio > 100%	2.42 (1.08 ~ 5.41)	**0.03**	0.11
	Sleep time	1.51 (1.06 ~ 2.14)	**0.02**	0.14
	Location			
	Anterior circulation	1		
	Posterior circulation	2.21 (0.85 ~ 5.71)	0.10	0.23
	Both	3.50 (0.43 ~ 28.49)	0.24	0.28
Depression	Gender (Female)	1.51 (0.64 ~ 3.59)	0.35	0.35
	Sleep time	1.62 (1.14 ~ 2.29)	**0.007**	**0.02**
	^2^Ratio > 100%	1.80 (0.80 ~ 4.03)	0.15	0.23

^1^ICVD represents “Ischemic cerebrovascular disease”, ^2^Ratio represents “Ratio of treatment cost to annual household income”

## Discussion

A pattern of significant psychological impairment was found to be associated with an identified but untreated UIA [5, 16, 17]. For example, Towgood K et al. [16] reported that 36% of untreated UIA patients presented with a pattern of significant psychosocial impairment 6 months post-treatment. Su SH et al. [5] reported that 84% of patients were found to have mild to severe anxiety 1 year after discovering the UIA. In addition, even 5 years after the detection of an UIA, 39% of patients were mildly to severely depressed, and 32% of patients had mild to severe anxiety. After an aneurysm is detected, many patients presented with complex psychological changes due to confusion regarding disease-related knowledge and sudden changes in their physical roles and physical functions, thus causing anxiety or depression symptoms.

Studies reported that patients with a longer duration since treatment typically showed lower anxiety and depression levels than those with a shorter duration since treatment [5, 10]. This phenomenon indicated that patients may still feel anxiety or depression for a short period of time after treatment because they fear bleeding or recurrence of the aneurysm, but these symptoms improve over time. Nevertheless, in this study, there was no significant difference in the incidence of anxiety and depression between the two groups stratified by the median duration since treatment (30 months), which may suggest that the anxiety and depression of these patients did not significantly decrease over time. In addition, several studies have suggested that a considerable number of UIA patients who undergo microsurgery or endovascular treatment suffer from anxiety or depression. For example, Li Y et al. [10] reported that 18.2% (*n* = 8) and 27.3% (*n* = 12) of the clipping group had anxiety and depression, respectively, and 17.6% (*n* = 13) and 24.3% (*n* = 18) of the coiling group had anxiety and depression, respectively. Solheim et al. [11] reported that 26.9% (*n* = 7) and 19.2% (*n* = 5) of the open surgery group suffered from anxiety and depression, respectively, and 31.6% (*n* = 6) and 10.5% (*n* = 2) of the endovascular coiling group suffered from anxiety and depression, respectively. Unfortunately, the results of these studies are limited by many factors, such as small sample sizes, different assessment instruments and patient cooperation.

Currently, endovascular treatment is considered a first-line strategy for UIAs because of its superiority to microsurgical clipping in terms of both morbidity and mortality [9, 18]. However, studies on anxiety and depression in UIA patients treated with endovascular intervention are rare and controversial. In addition, the risk factors for anxiety and depression in these patients need to be identified. This large cross-sectional study took place over an average period of 30.67 ± 8.6 months and assessed the anxiety and depression outcomes in UIA patients who underwent endovascular treatment. In our study, we found that 34 (17.0%) patients suffered from anxiety, and 31 (15.5%) suffered from depression, meaning that a certain number of patients were still in a state of psychological impairment after an average recovery period of 30.67 ± 8.6 months. The multivariate analysis results indicated that shorter sleep times were significantly associated with anxiety and depression. Thus, we recommend that patients should ensure adequate sleep and a healthy lifestyle. Treatment costs exceeding the annual household income and shorter sleep times was significantly associated with anxiety, although this was not statistically significant after FDR correction for *P*-values. Excessive financial burden tends create a state of anxiety. Therefore, corresponding policies and measures should be implemented to relieve the financial burden on these patients.

The main limitation of this study was its retrospective design. The anxiety and depression of these patients was not assessed before treatment, so it is not possible to compare anxiety and depression before and after treatment. Although our follow-up rate (90.0%) and questionnaire response rate (74.1%) are high and impressive, we still cannot ignore the existence of selection bias. In addition, the HADS is widely used for patients to provide a subjective assessment of depressive and anxiety symptomatology rather than as a standardized criterion for the diagnosis of clinical depression and anxiety, which may result in false-positive results. It is necessary to conduct a prospective and multicenter study in the future.

## Conclusion

Our current findings demonstrated that the prevalences of anxiety and depression in UIA patients treated with endovascular intervention were 17.0 and 15.5%, respectively. Shorter sleep times were significantly associated with depression. Our findings provide evidence for the clinical and psychological management of these patients.

## Data Availability

The datasets used and/or analyzed during the current study are available from the corresponding author on reasonable requests.

## Abbreviations

UIA: Unruptured intracranial aneurysm
HADS: Hospital Anxiety and Depression Scale
SAH: Subarachnoid hemorrhage
DSA: Digital subtraction angiography
MRA: Magnetic resonance angiography
CTA: Computed tomography angiography
mRS: Modified Rankin Scale
